# Risk network analysis and simulation research in municipal engineering projects contracted by China in Saudi Arabia

**DOI:** 10.1038/s41598-025-32219-z

**Published:** 2026-01-07

**Authors:** Rami Talal T Alotaibi

**Affiliations:** Faculty of Infrastructure Engineering, Dalian University of Technology, Dalian, 116024 Liaoning China

**Keywords:** General contracting, Municipal engineering project, SNA method, Risk relationship network, Risk control strategy, Health occupations, Risk factors

## Abstract

This study develops a risk network model using social network analysis (SNA) to address the complex interdependencies among risk factors in municipal engineering projects contracted by Chinese firms in Saudi Arabia. The four-type risk classification framework (Types I-IV) was introduced to categorize risks based on their network positions and characteristics. A case study of a large-scale municipal project was conducted, and the results showed that 34 risk factors were classified into 4 Type I risks, 2 Type II risks, 17 Type III risks, and 11 Type IV risks. Based on the network analysis, tailored preemptive and post-emergency response strategies were formulated for each risk type. Furthermore, a system dynamics simulation was employed for scene analysis of the proposed strategies. The simulation results demonstrated a significant improvement, with the project completion rate increasing from 77 to 90.5% after implementing the targeted risk responses. This work provides a systematic approach for Chinese contractors to understand risk propagation mechanisms and select optimal control strategies, thereby enhancing project success in the Saudi Arabian market.

## Introduction

 Saudi Arabia is an important overseas market for China’s construction industry in the Middle East. In recent years, as the cooperation between China and Saudi Arabia has deepened, an increasing number of Chinese construction companies have begun implementing engineering projects in Saudi Arabia. These projects span various fields, such as oil and gas, communication, industry, and municipal engineering. According to statistical data, in the first half of 2023 alone, the total contract value signed by Chinese companies in Saudi Arabia exceeded 5.5 billion US dollars^1^. With the continuous promotion of ecological construction and the modernization of transportation systems in Saudi Arabia, there will inevitably be more engineering projects, which represents a great opportunity for Chinese enterprises. However, companies worldwide have turned their attention to the Saudi Arabian market, and Chinese companies are facing significant competitive pressure^2^.

Risk management is a critical task in general contracting projects. Because of the uncontrollable and unavoidable nature of risks, it is crucial to develop reasonable risk prevention and remedial measures after a risk occurs^3^. Both the owner and the general contractor hope to solve the various risks that arise in the project at minimum cost^3^. This requires an accurate analysis of all possible risk factors and their interrelationships and consequences before the project starts, which is the main problem that this study aims to solve. For the general contracting unit, the ability to prepare a list of risk factors and formulate risk control measures largely determines market competitiveness. Therefore, the general contracting unit requires a scientific risk analysis method, which is the focus of this study.

For general contracting units in China, owing to significant differences in the natural and social environments of Saudi Arabia and China, Chinese companies face more diverse and complex risks when undertaking projects in Saudi Arabia^4^. The interconnected network of risk factors creates significant difficulties and pressure for project risk control. Therefore, for Chinese contractors, it is extremely important to comprehensively and reasonably summarize the potential risk factors that may arise in Saudi Arabian construction projects, evaluate the mutual influence and consequences of each risk factor, and develop effective risk prevention measures to improve profits and competitiveness^5^. While existing research has identified numerous risk factors in construction projects within Saudi Arabia, the majority of studies adopt static, isolated approaches centered on checklist-based inventories. These methods fall short of uncovering the systemic interdependencies and cascading effects among risk factors, which is critical for formulating effective risk mitigation strategies. This limitation is particularly pronounced in international EPC projects, where the inherently networked nature of risks is a defining characteristic. To bridge this gap, this study aims to address the following research questions:

1. How can the complex interdependencies among risk factors in international EPC projects be systematically modeled and analyzed?
2. What is a robust typology for classifying project risks based on their structural attributes within a risk network?
3. How effective are the tailored risk response strategies, derived from the network-based typology, in improving project outcomes?

## Literature review

The literature review consists of two parts: significant risks and risk methodology assessments. In terms of significant risks, the literature selected for this study mainly focused on the topic of risk analysis in Saudi Arabia’s engineering construction, and the project should be a general and common project, with special equipment and functional projects not considered. The literature review covered a period of 12 years. In terms of the risk methodology assessment, this study mainly selected relevant literature on the application of SNA methods to engineering risks. The literature on the application of SNA in other fields was not considered, and the literature period was within 12 years.

### Significant risks

The complex geological conditions in Saudi Arabia pose a major engineering risk for construction units. Almadani^6^ conducted an experimental analysis of engineering sites in central Saudi Arabia, and the results showed that the distribution of underground rocks was uneven. In this case, construction units must crush or replace rocks when performing foundation treatment to obtain a stable bearing layer, which results in additional foundation treatment costs. Simultaneously, uneven rocks may lead to changes in the foundation form from natural to piled foundations to solve the problem of uneven bearing layers. This change poses risks to project schedules and costs. The construction of pile foundations is time-consuming and incurs higher costs^7^. Adverse weather conditions are an important source of project risk. The extreme hot weather in Saudi Arabia every year not only affects the efficiency of workers and equipment, leading to longer construction periods, but may also cause workers to suffer from sunburn or illness, resulting in additional employment costs^8^.

Cultural differences are another major risk faced by overseas contractors in Saudi Arabia, reflected in various aspects, such as design, personnel arrangements, and language communication. In terms of design, Mohammad^9^ pointed out that foreign design institutes may not be able to come up with solutions that are in line with the actual situation in Saudi Arabia, owing to a lack of experience, and they may make mistakes, such as ignoring local design specifications and requirements. These erroneous design solutions inevitably lead to design rework and affect project schedules. In terms of personnel arrangements, many projects contracted by foreign companies still require the employment of local Saudi labor, which may lead to potential risks. Wei^10^ noted that local workers in Saudi Arabia have special work and rest habits and may need to take breaks or reduce their work intensity during Ramadan. However, many foreign contractors do not pay sufficient attention to local workers in Saudi Arabia, which can easily lead to dissatisfaction and the inability to complete work on time. In serious cases, it can also cause opposition from local society. Language differences are also a major reason for project risks. Almutairi^11^ believed that language differences might lead to communication difficulties between general contractors and subcontractors, especially when the general contractor directly issues orders to local workers in Saudi Arabia. Local workers may not fully understand the received orders, resulting in incorrect work behavior, which may lead to rework. Moreover, some professional terms have specific Arabic expressions, such as special materials and equipment models. If a general contractor is unfamiliar with Arabic, it may cause misunderstandings.

Material supply is another major risk faced by engineering projects in Saudi Arabia. Owing to design habits and cooperative relationships, foreign contractors are more willing to transport materials domestically rather than purchase them locally in Saudi Arabia. Cross-border transportation requires overcoming a series of problems such as weather, imports, and exports. Once a problem occurs in a certain transportation link, there may be a phenomenon of work stoppage owing to material shortages on site^12^. Abdullatif^13^ noted that unexpected events can affect a project’s material supply. For example, in the recent COVID-19 epidemic, the imported materials must undergo strict inspection, and it is likely that the materials will not be delivered to the site owing to unqualified inspection, which will lead to the risk of material shortage in the project. During project advancement, there may be a need for more steel bars and other materials owing to onsite design changes. In this case, domestic transportation may take too long, and there may be no substitute materials available locally in Saudi Arabia, leading to delays in the project schedule^14^. In addition, Esam^15^ proposed that, owing to the uncertainty of project functionality and owner preferences, there may be order revisions during project implementation, which means adjustments to transportation plans and changes in material inventory. These revisions may affect construction progress and result in additional procurement and transportation costs.

Based on the above analysis, existing research has explored the risks in Saudi Arabian engineering projects from various perspectives and has achieved rich results. However, few studies have been conducted on the risks of projects undertaken by Chinese contractors in Saudi Arabia. Moreover, projects undertaken by Chinese contractors have unique characteristics, and the cases and experiences of other countries are not fully applicable. Based on this, this study starts from the perspective of Chinese contractors, establishes a risk factor list and risk relationship network through project data investigation and expert interviews, analyzes the impact of risks, and formulates response measures to provide a basis for Chinese contractors to manage and control project risks. Table 1 summarizes common risk factors found in the literature.

**Table 1:** General risk factors in traditional municipal engineering projects identified from literature review.

	Risk factor	Relevant literature(s)
Technical & design	Inadequate site investigation	^6,7^
	Design errors or omissions	^9^
	Unfamiliarity with new technologies and materials	^10^
Management & organizational	Poor communication and coordination among parties	^11,16^
	Inadequate project planning and scheduling	^5^
	Insufficient experience and capability of contractors	^3^
External & environmental	Adverse weather conditions	^8^
	Unfavorable geological conditions	^6,7,18^
	Price fluctuations of materials	^14^
Legal & regulatory	Complex permit and approval procedures	^13^
	Changes in laws and regulations	^14^
Stakeholder & social	Public opposition or social pressure	^4^
	Land acquisition difficulties	^5^

### Application of SNA in risk management of construction project

Social Network Analysis (SNA) is derived from mathematics and social humanities to analyze group interaction behaviors. It is widely applied in risk identification and domestic and international warnings. For example, SNA is used for risk analysis of social network interpersonal relationships^16^ and risk analysis of epidemic infection and spread^17^. In recent years, the SNA method has been increasingly applied in the field of engineering management and has played a significant role in the risk analysis and control of engineering projects. Zelenakova^18^ established a risk relationship network model for water treatment engineering based on SNA and revealed the potential risks and mechanisms of action in water treatment engineering. Chen et al.^19^ established a risk relationship network model for urban underground engineering in China and identified the core risk factors and risk diffusion activities in underground engineering, thereby providing risk control measures. Huang et al.^20^ used the SNA method to analyze the correlation between risk factors in green building projects and the impact of risk occurrence time on the degree of risk harm and proposed risk prevention strategies. Xu et al.^21^ established a risk evolution model for urban river ecological governance projects based on SNA, revealing the causes and development processes of risks in these projects and providing a reference for risk control. Zhu and Liu^22^ established a stakeholder relationship network for green building projects based on SNA, analyzed the relationships between different stakeholders and their impact on the project, and proposed project governance methods based on the results. Chao et al.^23^ analyzed the risk interaction behavior of large-scale engineering projects using the SNA method. The core risks of the project were extracted through network feature analysis, and the impact weights of the core risks on other risks were calculated. Finally, corresponding risk prevention and control measures were proposed. Wang et al.^24^ used the SNA method to evaluate the safety risks in ocean bridge construction projects. By establishing a risk network topology model, they effectively identified the key risks and critical triggering points in ocean bridge construction. The results provide support for the prevention of safety risks. Dehdasht et al.^25^ used the SNA method to identify and analyze the key driving factors in construction projects and designed optimization measures based on the action paths of the key driving factors to improve the overall efficiency and sustainability of construction projects.

From the above results, it can be seen that the SNA method has been widely applied in the risk analysis of engineering projects. SNA theory emphasizes the order and logical relationships of nodes in a network. This allows an intuitive and concrete display of the interactions and influences between nodes through directed edges. As a result, SNA is an ideal method for solving and managing complex network problems, particularly for sorting and analyzing risk relationships in general international contracting projects. Although SNA has been widely applied in various engineering projects (such as underground engineering, green buildings, large-scale infrastructure, etc.), most studies only rely on expert scoring to determine the strength of relational ties, rendering the objectivity and generalizability of their conclusions subject to further validation. Furthermore, research that systematically categorizes risk nodes based on network metrics and explicitly links these categories to differentiated response strategies remains underdeveloped. To address these gaps, this study not only employs SNA but also incorporates system simulation to achieve scene analysis of the strategies derived from the network analysis. Consequently, we propose an integrated “network analysis-classification-strategy formulation-simulation scene” framework, thereby providing a meaningful extension and contribution to the existing body of SNA application research.

### Research gaps and contributions

Although existing research is rich, there are still some gaps that need to be filled, including the following two points: First, contractors from different countries have varying experiences in dealing with project risks, and the degree of risk impact differs. Therefore, research must be conducted considering the characteristics of general contractors. In the existing literature, references^6^ to^15^ cited above are all from countries such as Germany and Saudi Arabia, and few studies have been conducted from the perspective of Chinese contractors. As a major infrastructure country, China has undertaken several projects in Saudi Arabia. Therefore, risk research based on the perspective of Chinese contractors is necessary and should be supplemented. Second, although the SNA method has a good effect on the analysis of risk relationships in engineering projects, existing studies^19–25^ all use expert scoring methods to determine the strength of the relationships between network factors and provide relevant conclusions. Due to the unverified nature of these conclusions, their results are highly dependent on expert experience. However, project risks themselves have significant uncertainty, and relying solely on subjective evaluations can easily deviate from the actual situation. Therefore, after drawing conclusions, effective verification methods should be adopted and enriched.

To solve these problems, the following study was conducted. First, statistical data on Chinese general contracting projects in Saudi Arabia were analyzed and summarized, and the key risk factors faced by Chinese contractors were selected. At the same time, management personnel from Chinese general contracting units and experts from Chinese universities were invited to form an expert group to discuss and evaluate risk factors and obtain a list of risk factors. The risk factor list established in this study has clear characteristics of Chinese contractors and is suitable for risk analysis of engineering projects of Chinese general contracting units in Saudi Arabia. Second, a system simulation model was established to achieve scene reproduction of the network analysis. By simulating a specific engineering example, project schedule progress under three different scenarios was simulated, demonstrating the effectiveness of the proposed risk prevention and control measures.

## Materials and methods

### Risk relationship network model

**Fig. 1:**
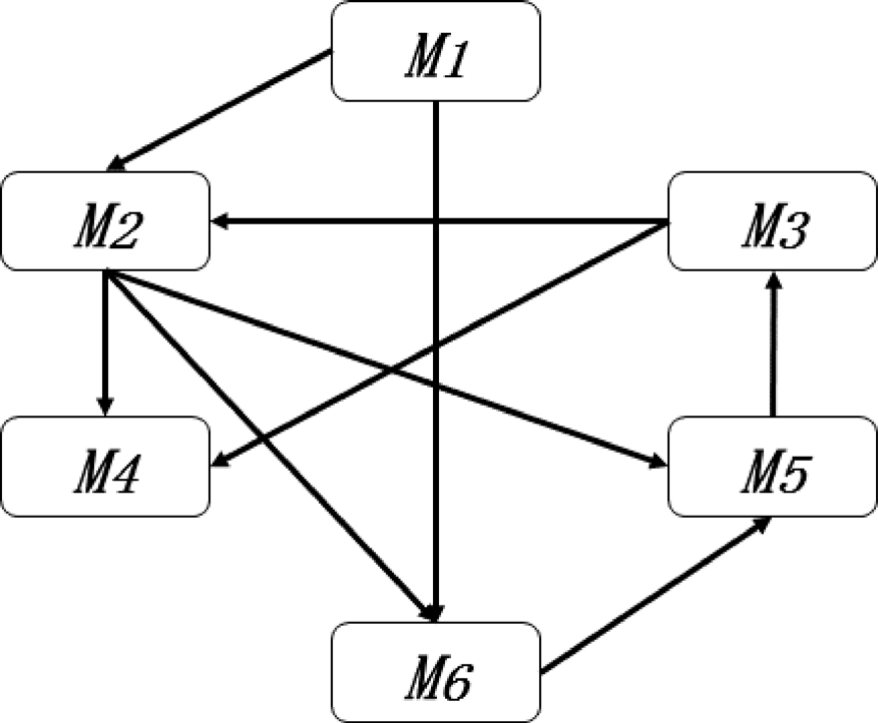
Schematic diagram of risk relationship network.

The risk relationship network in the SNA theory is composed of nodes and directed edges, where nodes represent risk factors and directed edges represent the impact relationship of each risk factor on other risk factors. In actual engineering projects, the impact of each risk factor on other risk events is primarily related to the logical sequence of processes, constraints, and materials. This study used Likert scale (0 ~ 5) to determine the impact of the risk factors. This means that if one risk factor had no direct impact on another risk factor, it was scored as zero point. Otherwise, it was scored from one to five point to indicate the strength of the impact, where five point represented extremely strong impact and one point represented extremely weak impact. The risk factor relationship matrix can be obtained by sequentially calculating the impact relationships between various risk factors. To illustrate the correspondence between the risk factor relationship matrix and the risk relationship network, it is assumed that the risk set of a certain project consists of six risk factors: *M*_*1*_, *M*_*2*_, *M*_*3*_, *M*_*4*_, *M*_*5*_, *M*_*6*_. The risk relationship matrix obtained from expert scoring is presented in Table 2. The risk relationship network shown in Fig. 1 can be obtained based on the risk relationship matrix.

**Table 2:** Risk factor relationship matrix.

	*M*_*1*_	*M*_*2*_	*M*_*3*_	*M*_*4*_	*M*_*5*_	*M*_*6*_
*M*_*1*_	0	3	0	0	0	1
*M*_*2*_	0	0	0	2	3	4
*M*_*3*_	0	5	0	1	0	0
*M*_*4*_	0	0	0	0	0	0
*M*_*5*_	0	0	2	0	0	0
*M*_*6*_	0	0	0	0	2	0

### Risk factor categories

This study categorizes risk factors into four types based on their locations and characteristics within the network, considering their different timings, scopes of impact, and handling methods.

**Fig. 2:**
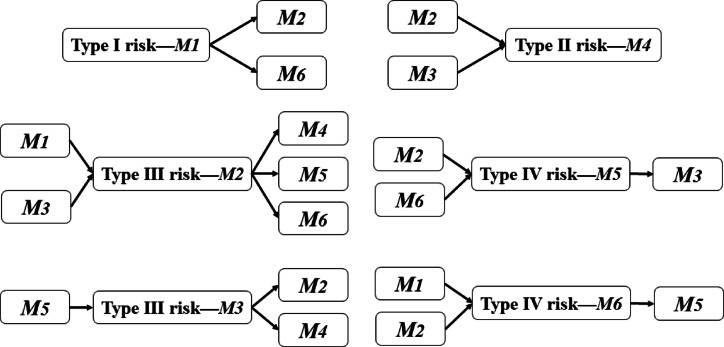
Schematic diagram of risk factor categories.

Type I risk refers to the primary node in the risk chain within the network of risk relationships that exerts a significant influence on other risk factors while being minimally affected. According to the risk relationship matrix in the previous section, *M*_*1*_ belongs to Type I risk, as depicted in Fig. 2.

Type II risk: This is the terminal node in the risk chain within the network of risk relationships, which is heavily impacted by other risk factors but does not influence them. According to the risk relationship matrix in the previous section, *M*_*4*_ belongs to the Type II risk category, as illustrated in Fig. 2.

Type III and Type IV risks: These are nodes that are not only affected by other risk factors but also influence them. This distinction lies in the fact that the impact of Type III risks is lower than that of external risks, whereas the impact of Type IV risks is higher than that of external risks. According to the risk relationship matrix in the previous section, *M*_*2*_ and *M*_*3*_ belong to Type III risk, while *M*_*5*_ and *M*_*6*_ belong to Type IV risk, as shown in Fig. 2.

### Network analysis indicators

According to SNA theory, this study selects degree centrality as the indicator to analyze risk factors, and the corresponding indicators are adopted based on the definition of the four types of risks. For Type I and Type II risks, degree centrality was used for identification, where the in-degree of Type I risk was 0 and the out-degree of Type II risk was 0. In the SNA method, the in-degree represents the level of influence of the preceding factors on the node, whereas the out-degree represents the level of influence on subsequent factors^26,27^. From a project management perspective, early intervention and prevention should be implemented for factors with a low in-degree and high out-degree to reduce the likelihood of risk factor occurrence and control the scope of the risk impact28,^29^. Emergency management should be carried out for factors with a high in-degree but low out-degree to reduce the impact of the risk events themselves^30,^^31^.

Type III and Type IV risks: these are intermediary nodes that are both affected by and affect other risk factors (i.e., both in-degree and out-degree are greater than zero). The critical distinction lies in whether a risk factor is a net influencer or a net receiver of impacts within the network^32,^^33^. To quantify this, we introduce two normalized indices:

Relative weighted out-degree value measures the proportion of a node’s total outgoing influence relative to the sum of all edge weights in the network^34^. It is calculated as the weighted out-degree of node *i* divided by the sum of all elements in the weighted risk relationship matrix *A*^35^, according to Eq. (1):

1 $${C_{w - out}}\left( i \right)=\frac{{\sum\limits_{{j \in N}} {{a_{ij}}} }}{{\sum\limits_{{i \in N}} {\sum\limits_{{j \in N}} {{a_{ij}}} } }}$$

In Eq. (1):

${a_{i,j}}$——The impact intensity of risk *i* on risk *j*;

Relative weighted in-degree value measures the proportion of a node’s total incoming influence relative to the sum of all edge weights in the network. It is calculated as the weighted in-degree of node *i* divided by the sum of all elements in the weighted risk relationship matrix *A*^36^, according to Eq. (2):

2 $${C_{w - in}}\left( i \right)=\frac{{\sum\limits_{{j \in N}} {{a_{ji}}} }}{{\sum\limits_{{i \in N}} {\sum\limits_{{j \in N}} {{a_{ij}}} } }}$$

A risk factor is classified as Type III if its relative weighted out-degree value is greater than its relative weighted in-degree value. This indicates that its role as an originator of impacts outweighs its role as a receiver. Conversely, a risk factor is classified as Type IV if its relative weighted in-degree value is greater than its relative weighted out-degree value, indicating it is more susceptible to being affected by the network than it is capable of affecting others. This method provides a theoretically grounded, reproducible, and non-arbitrary means of distinguishing between these two complex risk types.

### System simulation method

SNA can effectively display the correlation and degree of impact of various factors on a risk relationship network. However, its indicator weight depends on the subjective judgment of experts and scholars, which may not translate directly into engineering practices. Therefore, after constructing a risk relationship network, it is important to use appropriate methods to achieve scene reproduction and analysis. A commonly used method for this purpose is system simulation. This method involves establishing a system simulation model based on the correlation and influence coefficients of risk factors in the network. By setting parameters to achieve risk warning and emergency response, the simulation results can be compared with engineering examples to determine the degree of fit between the risk network and actual situations^37^.

**Fig. 3:**
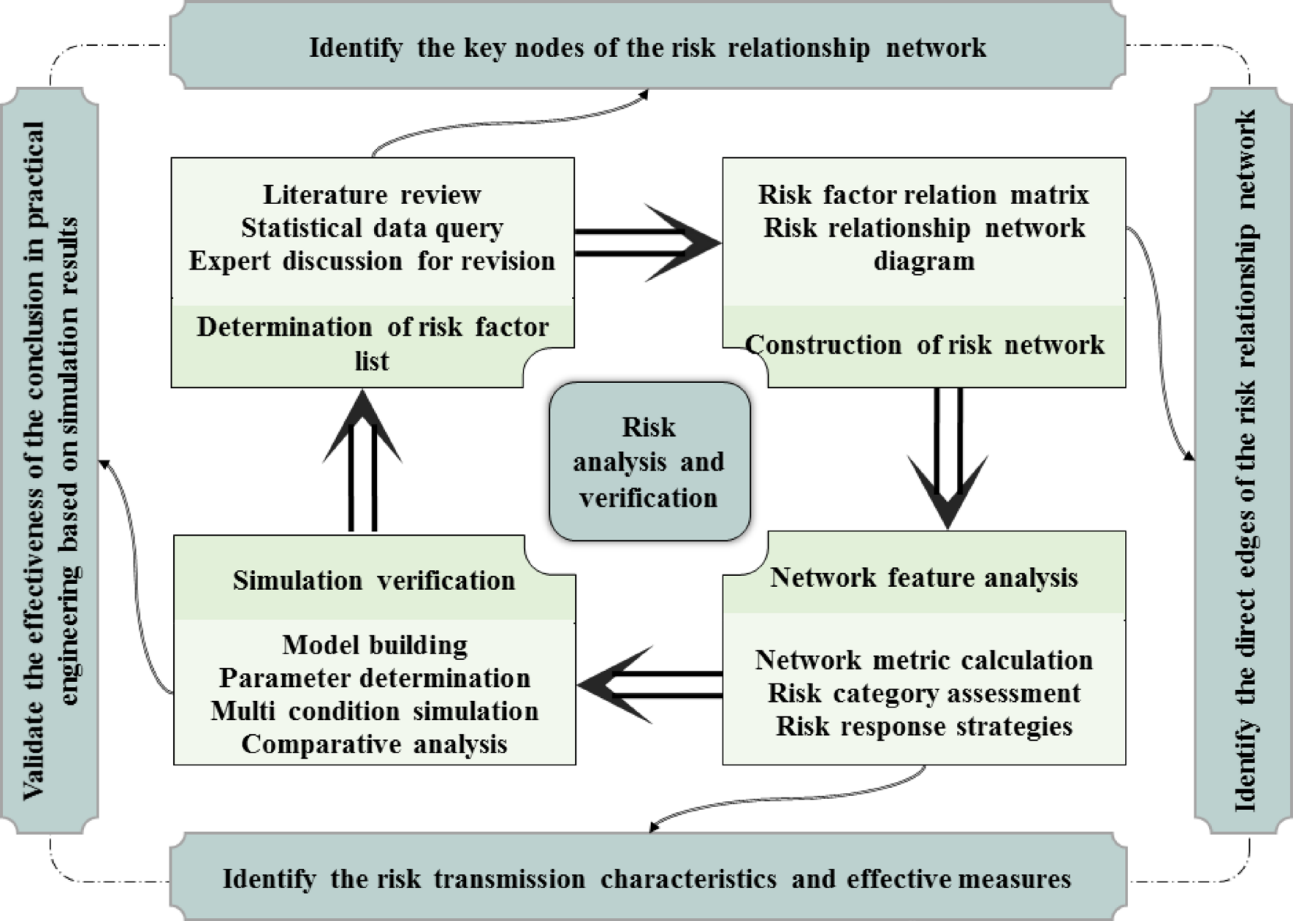
Technical roadmap.

The technical roadmap of this article is shown in Fig. 3.

## Results and discussions

As a case study, we examine a large-scale municipal engineering project contracted by a Chinese contractor in Saudi Arabia. It analyzes the risk factors of the project, discusses the characteristics of its relationship network, and provides countermeasures. This project was a general contracting project using the EPC mode and was located in the northern border province of Saudi Arabia. The project investor was the government of the northern border province, and the project included the construction of two urban roads and supporting underground pipelines, as well as greening and lighting around the roads. The project was designed by a large state-owned design institute and contracted by a large general contracting enterprise in China. The subcontracting units included 12 Chinese and six Saudi companies. Except for the concrete and steel purchased locally, all other components used in the project were provided domestically in China. The total investment in the project was USD 340 million, and the project period was 60 months. The project was divided into three stages: early organization and coordination, engineering design and construction, and maintenance and operation. Owing to the involvement of a large number of interest groups in the project, as well as the large amount of work and capital investments, the overall level of risk management was relatively average.

### Construction of risk relationship network

This study mainly employed data reviews, interviews, and expert evaluations to identify the risk factors in the project and create a relationship network. Initially, the general risk factors of traditional municipal engineering projects were summarized through a literature review, followed by a preliminary risk list. We then collected and consulted various project materials, including project proposals, feasibility study reports, preliminary designs, survey reports, progress plans, bidding documents, construction schedules, supervision and acceptance records, and other relevant documents, to adjust and supplement the risk list.

The preliminary risk list, derived from the literature, was subsequently refined and supplemented through a thorough review of specific project documentation obtained for the case study project. The types of documents consulted and the typical risk-related information extracted from each are outlined below:

Project Proposal & Feasibility Study Report: These documents were analyzed to identify risks related to project scope definition, initial budget accuracy, and potential high-level stakeholder concerns. (e.g., Risks such as *N*_*2*_: Difficulty in unifying engineering construction standards).

Preliminary Design Documents & Survey Reports: These were scrutinized for risks associated with design assumptions, site suitability, geotechnical uncertainties, and the condition of existing underground utilities. (e.g., Risks such as *N*_*3*_: The survey result file is not standardized, *N*_*5*_: Unclear situation of existing underground facilities, *N*_*18*_: Frequent occurrence of complex geological conditions).

Bidding Documents & Contracts: These were reviewed to understand risks pertaining to contract clarity, responsibility allocation between parties, penalty clauses, and insurance requirements. (e.g., Risks such as *N*_*28*_: Unclear handover and management responsibilities).

Construction Schedules & Progress Reports: Analysis of these records helped identify risks concerning schedule adherence, resource allocation efficiency, and the impact of unforeseen delays. (e.g., Risks such as *N*_*24*_: Uneven progress and quality in various sections).

Supervision & Acceptance Records, Meeting Minutes: These documents provided insights into risks emerging during construction, such as quality non-conformances, safety incidents, communication breakdowns, and change order management. (e.g., Risks such as *N*_*14*_: Inadequate construction safety management, *N*_*16*_: Lack of communication and exchange between various units, *N*_*20*_: Inadequate supervision by supervisors).

Subsequently, to ensure the reliability and validity of the risk factor identification and relationship scoring, a structured expert elicitation process was employed. An expert panel was meticulously formed, comprising 12 specialists with extensive experience and expertise relevant to international construction projects, particularly in the Saudi Arabian context. The panel’s composition was as follows:

Four project managers from leading Chinese general contracting enterprises, each with over 10 years of experience managing large-scale infrastructure projects in the Middle East.

Three senior managers from local Saudi Arabian construction firms, providing crucial insights into the regional regulatory environment, labor practices, and material supply chains.

Two academic scholars from Saudi universities specializing in construction engineering and project risk management.

Three professors from Chinese universities whose research focuses explicitly on international engineering risk analysis and Social Network Analysis applications.

The selection of the 12-expert panel was deliberate to ensure the quality and reliability of the data collected. The sample size is consistent with the number of experts typically involved in similar qualitative and expert-opinion-based risk analysis studies in construction management^19,22,23^. More importantly, the composition of the panel was designed to incorporate diverse yet highly relevant perspectives:

Practitioner Perspective (7 experts): Project managers from the general contractor and managers from local construction units provided first-hand, practical insights into the operational challenges and risk dynamics encountered in Saudi Arabia. Their extensive experience ensured that the identified risks were relevant to real-world scenarios.

Academic & Research Perspective (5 experts): Scholars from both Chinese and Saudi universities, specialized in engineering risk management, contributed theoretical rigor and a cross-cultural understanding of risk factors. Their involvement helped in structuring the risk list and the relationship assessment methodology.

This combination of practical expertise and academic knowledge ensured a comprehensive and balanced identification and evaluation of risks. The panelists possessed deep understanding of both the technical aspects of municipal engineering and the socio-cultural context of Saudi Arabia, making them exceptionally well-suited for the purpose of this research. Therefore, the selected sample, though not large in absolute terms, is considered robust and appropriate for achieving the research objectives through expert elicitation.

To ensure a systematic and comprehensive identification of risk factors, a semi-structured interview protocol and expert evaluation framework were developed and adhered to. The guiding questions and discussion points during the interviews and expert sessions primarily revolved around the following themes:

1. Based on the preliminary risk list derived from the literature review, what are the perceived likelihood and impact of these risks in the context of Chinese-contracted municipal projects in Saudi Arabia?
2. Are there any significant risks missing from the preliminary list, considering the specific project phases (early organization, design & construction, maintenance & operation)?
3. Considering the unique context of China-Saudi cooperation, what are the critical risks arising from cultural differences, regulatory environments, supply chain complexities, and technical standards alignment?
4. Could you describe any specific risk events or near-misses encountered in similar projects and their root causes?
5. How do you perceive the interrelationships between different risk factors? For instance, could the occurrence of Risk *N*_*1*_ typically trigger or exacerbate Risk *N*_*2*_?

The experts discussed these open-ended questions on the basis of preliminary risk list, then some suggestions were proposed to modify the risk list. Finally, a final risk list that included 34 risk factors across three project stages was determined, as shown in Table 3.

**Table 3:** Project risk list.

Stage number	Stage	Number	Risk factor	Number	Risk factor
1	Early organization and coordination	*N*_*1*_	Complex project approval procedures	*N*_*2*_	Difficulty in unifying engineering construction standards
		*N*_*3*_	The survey result file is not standardized	*N*_*4*_	Surrounding residents have resistance emotions
		*N*_*5*_	Unclear situation of existing underground facilities	*N*_*6*_	Insufficient ability to apply new technologies
		*N*_*7*_	Difficulty in land acquisition	*N*_*8*_	Unclear selection of materials and equipment
2	Engineering design and construction	*N*_*9*_	Lack of experience among designers	*N*_*10*_	Cost increase due to owner changes
		*N*_*11*_	Too many suppliers and chaotic management	*N*_*12*_	Material delivery delay
		*N*_*13*_	Construction personnel do not have relevant qualifications	*N*_*14*_	Inadequate construction safety management
		*N*_*15*_	Price fluctuations in the procurement market	*N*_*16*_	Lack of communication and exchange between various units
		*N*_*17*_	Insufficient quality control measures	*N*_*18*_	Frequent occurrence of complex geological conditions
		*N*_*19*_	Extreme weather affecting construction progress	*N*_*20*_	Inadequate supervision by supervisors
		*N*_*21*_	Unreasonable personnel allocation	*N*_*22*_	Local government and social pressure
		*N*_*23*_	Bias in laws and regulations	*N*_*24*_	Uneven progress and quality in various sections
		*N*_*25*_	Currency depreciation	*N*_*26*_	Language barrier
3	Later maintenance and operation	*N*_*27*_	Inadequate protection of finished products	*N*_*28*_	Unclear handover and management responsibilities
		*N*_*29*_	The mechanism for identifying and filling in deficiencies is not sound	*N*_*30*_	The operation and usage of each district are not synchronized
		*N*_*31*_	Poor management level of the construction unit	*N*_*32*_	Insufficient depth of project acceptance
		*N*_*33*_	Inadequate guarantee measures of project operation	*N*_*34*_	Insufficient production guarantee and optimization tracking

The expert group was invited to score the interrelationships of the risk factors, and the risk factor relationship matrix was obtained. Prior to the scoring sessions, all experts participated in a comprehensive briefing. This session detailed the study’s objectives, provided clear definitions of the 34 preliminary risk factors, and explained the 0–5 scoring methodology for establishing the risk relationship matrix. To mitigate potential group bias, the initial round of scoring was conducted anonymously using a structured questionnaire. Following this, a facilitated discussion was held to address significant discrepancies in scores. In cases where consensus was not reached after discussion, a second anonymous scoring round was conducted specifically for those contentious risk relationships. The final risk relationship matrix was derived by aggregating these scores, adopting relationships that were consistently identified by over 70% of the panel members. This rigorous, multi-stage process enhances the replicability and validity of the expert-derived data used to construct the risk network.

**Fig. 4:**
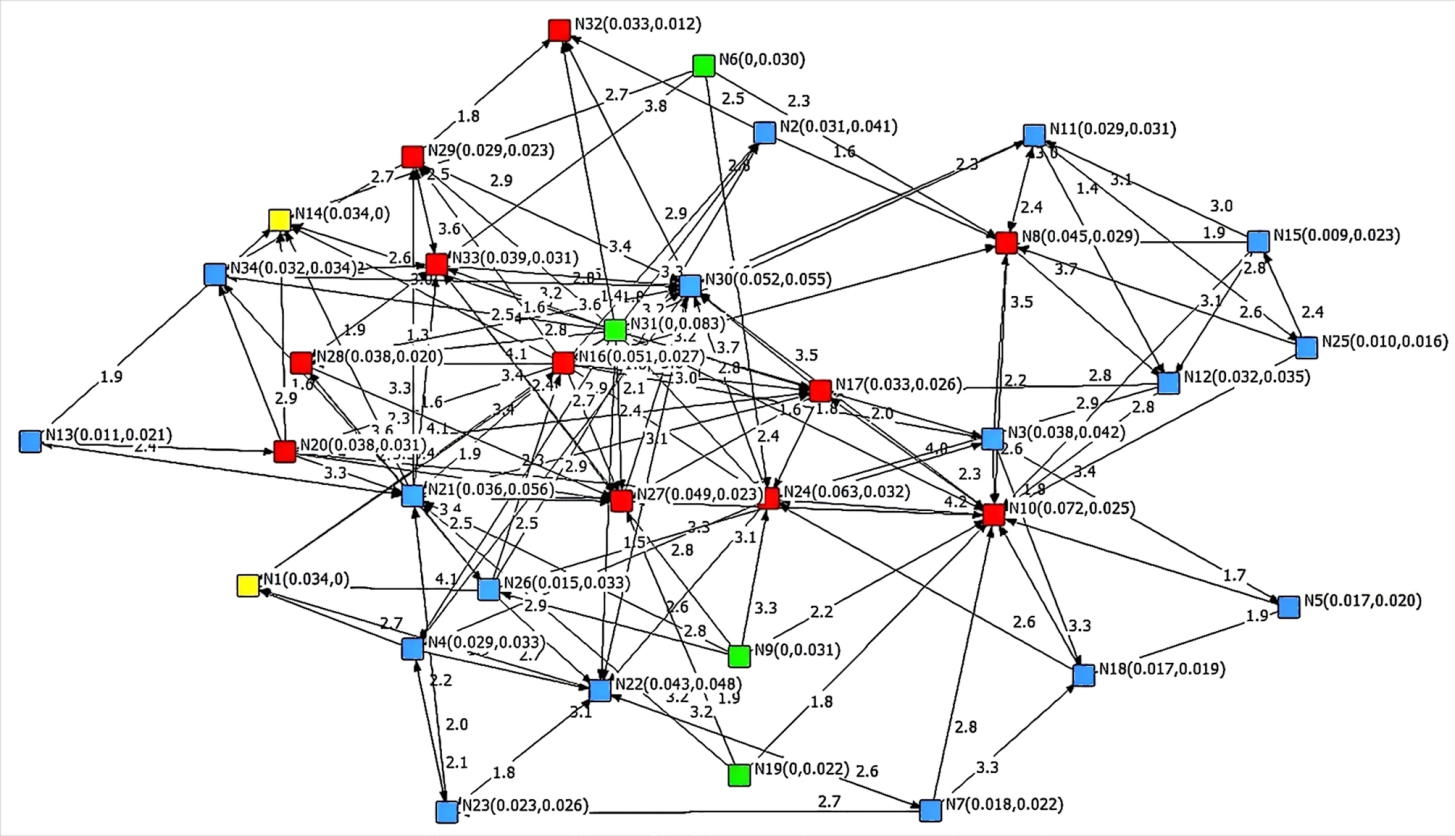
Risk relationship network diagram.

UCINET software specializes in network analysis with powerful computational analysis capabilities that can quickly and accurately provide users with the required network metrics and statistical data. UCINET version 6.560 was used for the analyses and calculations. The risk factor relationship matrix was input into the software in the form of an Excel spreadsheet. UCINET can read the number of rows and columns in the matrix, generate nodes, and then create directed edges between nodes based on the data in the matrix to generate a risk relationship network. After the risk relationship network was generated, the output data selected for relative weighted in-degree value, relative weighted out-degree value were obtained according to the research needs. UCINET automatically calculated and output the final results, and the calculation formula was the same as that used in this study. NetDraw software was built into UCINET, which enabled network visualization. By selecting NetDraw on the UCINET interface, UCINET converts the calculation results into the VNA format and inputs them into NetDraw software version 6.560 to draw the risk relationship network, as shown in Fig. 4.

In Fig. 4, nodes of different colors represent different types of risks. The green nodes represent Type I risk, the yellow nodes represent Type II risk, the blue nodes represent Type III risk, and the red nodes represent Type IV risk. The two numbers in parentheses after each node represent the relative weighted in-degree value and relative weighted out-degree value, respectively. The numbers marked on each edge in the network represent the risk impact intensity values.

### Risk relationship network analysis

The analysis indicators of each risk factor were calculated using the UCINET software, as shown in Table 4. In the table, relative weighted out-degree value is calculated according to Eq. (1), relative weighted in-degree value is calculated according to Eq. (2).

**Table 4:** Analysis results of risk factor Indicators.

Number	Degree centrality		Relative weighted in-degree value	Relative weighted out-degree value	Risk type
	in	out			
*N*_*1*_	5	0	0.034	0	II
*N*_*2*_	3	2	0.031	0.041	III
*N*_*3*_	3	4	0.038	0.042	III
*N*_*4*_	4	3	0.029	0.033	III
*N*_*5*_	1	2	0.017	0.020	III
*N*_*6*_	0	4	0	0.030	I
*N*_*7*_	2	4	0.018	0.022	III
*N*_*8*_	7	3	0.045	0.029	IV
*N*_*9*_	0	5	0	0.031	I
*N*_*10*_	13	3	0.072	0.025	IV
*N*_*11*_	3	4	0.029	0.031	III
*N*_*12*_	3	3	0.032	0.035	III
*N*_*13*_	1	3	0.011	0.021	III
*N*_*14*_	6	0	0.034	0	II
*N*_*15*_	1	4	0.009	0.023	III
*N*_*16*_	9	6	0.051	0.027	IV
*N*_*17*_	6	4	0.033	0.026	IV
*N*_*18*_	3	3	0.017	0.019	III
*N*_*19*_	0	3	0	0.022	I
*N*_*20*_	5	6	0.038	0.031	IV
*N*_*21*_	6	11	0.036	0.056	III
*N*_*22*_	7	8	0.043	0.048	III
*N*_*23*_	3	3	0.023	0.026	III
*N*_*24*_	11	4	0.063	0.032	IV
*N*_*25*_	1	3	0.010	0.016	III
*N*_*26*_	2	6	0.015	0.033	III
*N*_*27*_	9	3	0.049	0.023	IV
*N*_*28*_	6	3	0.038	0.020	IV
*N*_*29*_	4	4	0.029	0.023	IV
*N*_*30*_	11	10	0.052	0.055	III
*N*_*31*_	0	18	0	0.083	I
*N*_*32*_	4	2	0.033	0.012	IV
*N*_*33*_	7	5	0.039	0.031	IV
*N*_*34*_	6	5	0.032	0.034	III

### Risk response strategies

In the actual construction process, the manager relies solely on experience to deal with various risk factors. The final results showed that the effect was poor, and the engineering data indicated that the project cost and schedule were both affected to a certain extent. This section proposes targeted risk response strategies based on the characteristics of the risk factors and their position in the network analyzed earlier, aiming to improve the level of risk management. Based on the contents of this section, a list of strategies corresponding to the risk factors can be compiled and promoted for application in future projects, providing a reference for project managers to control risks more effectively.

#### Type I risk

Based on the findings in Table 3, four Type I risks were associated with this project. These include insufficient ability to apply new technologies, lack of experience among designers, extreme weather affecting construction progress, and poor management within the construction unit. Type I risks are the starting points in the risk chain; their occurrence impacts other nodes and triggers new risk events. To reduce the probability of occurrence and impact of Type I risks, the principle of prior control should be adopted.

This is a key construction project in Saudi Arabia, and the Saudi Arabian government has invested heavily in it, which involves many cutting-edge technologies and equipment. However, Chinese design and construction teams lack sufficient work experience, and their technical reserves are poor. Therefore, in the early stages of a project, the contracting unit should organize professional and technical personnel in design, construction, and other fields to receive training. This enables them to master the current industry norms and standards in Saudi Arabia and learn from the excellent technology and construction experience of past local projects. Saudi Arabia has a tropical desert climate that is prone to extremely high temperatures. Therefore, the contracting unit should develop a high-temperature warning mechanism and response plan in advance, and it is necessary to purchase heatstroke prevention facilities to avoid injuries to personnel and materials caused by high-temperature weather.

#### Type II risk

This project faced two types of risks: complex project approval procedures and inadequate construction safety management. Type II risks occur at the end of the risk chain and require post-event emergency measures to reduce their impact. For example, project managers should immediately implement emergency measures when construction safety management issues are identified. On one hand, it is necessary to immediately correct the problem, strictly punish the relevant persons, and carry out rectification immediately. Construction is not allowed to continue until safety standards are met to reduce the impact of safety issues and prevent them from escalating into more serious accidents. However, project managers should also organize safety training for all parties, strengthen safety awareness, and add safety measures such as deploying more safety officers and increasing onsite monitors to maintain the safety level within a controllable range and avoid the impact of safety issues on other aspects of the project. Simultaneously, eliminating the connection between risk factors and higher-level factors can help reduce the probability of risk occurrence^38^. For example, regarding the issue of complex project approval procedures, many departments in Saudi Arabia approve projects independently, and the construction content of municipal engineering involves multiple departments, such as government, transportation, and communication, making it difficult to achieve unified approval. Therefore, contractors can reasonably classify the approval materials. For the parts that can be independently approved, a declaration can be made first, and for the parts that require comprehensive approval, the approval time can be saved. This method reflects the risk-cutting behavior when materials that can be independently approved are separated; this part of the approval is no longer affected by the risk of complex approval processes.

#### Type III risk

This project involves 18 Type III risks, in which the principle of prior control should be adopted. Type III risks mainly include several aspects, namely an unclear site environment, unstable material supply, personnel allocation, language issues, and pressure exerted by local governments and residents. For general contractors, the required survey data should be proposed before the project starts, and the survey unit should conduct a comprehensive and detailed survey. After the survey is completed, the geological conditions and underground facility distribution around the site must be fully demonstrated to create favorable conditions for subsequent design and construction. Second, general contractors should have a thorough understanding of the local materials market in Saudi Arabia and purchase materials locally as much as possible. If domestic materials are required, transportation route planning should be performed in advance, and transportation time and volume should be determined. Moreover, local materials and domestic suppliers should be managed separately, and dedicated personnel should be arranged for coordination. Third, to address the issues of personnel allocation and language, contractors should move away from the rigid thinking of implementing projects in China. Therefore, it is necessary to develop a comprehensive implementation plan during the initial stages of a project. Furthermore, the environmental characteristics of Saudi Arabia and construction requirements of the owner should be analyzed to determine the personnel allocation plan. For instance, the work habits in Saudi Arabia differ from those in China. Local project participants have relatively short working hours and are unable to work during Ramadan. Managers should fully consider these factors and make appropriate arrangements for personnel. In addition, Chinese and Saudi workers should be managed separately, and sufficient translators should be hired to address language issues. Fourth, regarding the pressure exerted by local governments and residents, project managers must fully understand the local customs and traditions of Saudi Arabia during the project planning stage, particularly the many restrictions on non-Muslim personnel. Furthermore, project participants’ behaviors must be strictly regulated to prevent social instability.

#### Type IV risk

12 Type IV risks require emergency response measures to reduce their impacts. Type IV risks primarily include four aspects: owner change, poor communication between participating units, lax control of progress and quality, and insufficient operational and maintenance capabilities. First, in previous projects, when there was a change in the owner, the contractor usually needed to commission the design to be reworked and then procure and construct again based on the new design plan, which consumed considerable time and cost^39^. Therefore, to handle the risk of owner changes better, the contractor must communicate and negotiate with the owner, design party, and construction personnel to use surplus or easily procurable materials in Saudi Arabia to complete engineering changes. Efforts should also be made to utilize existing job types and project technologies to minimize the costs resulting from changes. Second, language barriers and cultural differences may also lead to poor communication. In previous projects, contractors were concerned about disputes arising from different understandings; therefore, they usually left it to local subcontractors in Saudi Arabia to manage on their own, which could easily cause errors and problems^40^. The project department should establish an effective information transmission channel to communicate the events that occur at the project site to various units. Regular plenary meetings should be organized to share information and experiences and foster a mutual understanding of strengths and weaknesses, which is crucial for cooperation. Additionally, difficult problems encountered during the project construction process should be discussed to reach a consensus among all parties for proper resolution. Third, in previous projects, when the construction scope was large and the construction process was complex, contractors were usually unable to supervise comprehensively and only discovered and rectified progress and quality issues when they had significant consequences^41^. To ensure project quality and progress effectively, project management personnel should promptly organize construction units and allocate personnel to reinforce the construction intensity of lagging blocks and non-conforming areas to unify progress. Moreover, intensified regulatory efforts should be made for blocks subcontracted to local construction personnel, providing technical and material support to standardize project progress. Fourth, in many early engineering projects, contractors withdrew from the site after completing project construction, and owners often encountered situations in which they were unable to take over the project owing to unfamiliarity with the project content^42^. Regarding this issue, the contractor should provide sufficient technical support to the owner, invite design experts, operation experts, etc. to provide technical guidance for the maintenance and commissioning of the project in the later stages, help the owner understand the project content, and make a reasonable transition.

### Simulation scenario analysis

In this section, we use a system simulation to achieve scenario analysis and determine the correlation between the risk factors and the effectiveness of the proposed response strategies.

#### System simulation model

**Fig. 5:**
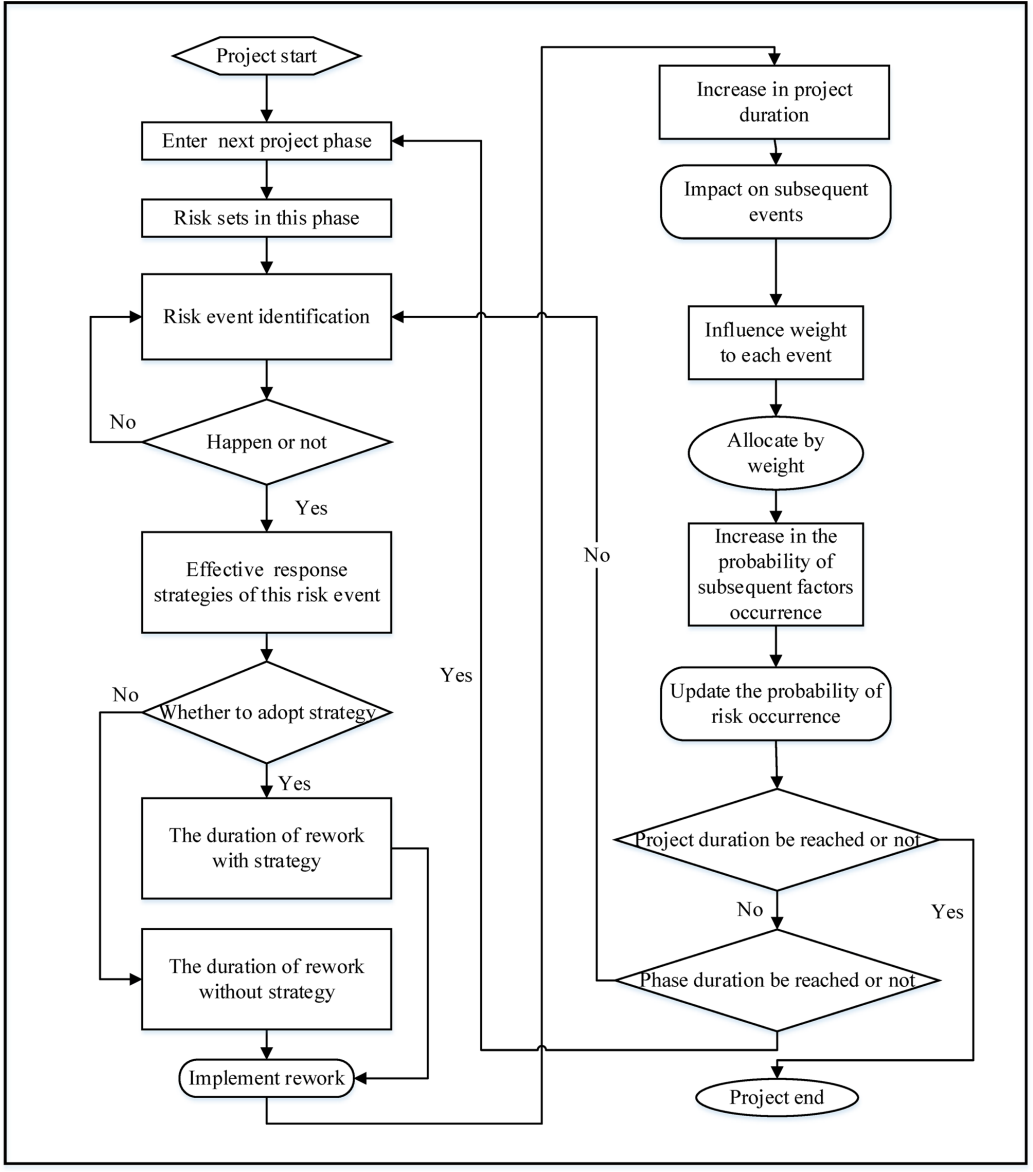
Simulation system flowchart.

In this study, a modeling method based on a construction sequence was used to create a system simulation model. Each major process includes several sub-processes, such as approval, surveying, design, procurement, construction, and product protection. The risk factors were mitigated through reworking. When a risk factor arises, the affected process is identified based on the nature of the risk factor, and the necessary rework duration is determined^43^. The rework duration can be divided into two situations based on whether a strategy has been adopted. The total rework duration was used as an evaluation indicator for the risk control strategy. The weighting method was used to determine the correlation between risks. The weight value of the impact of a single risk on the other risks was calculated based on the risk correlation matrix. When a certain risk occurs, the model divides its probability of occurrence into other factors affected by it according to the weight ratio. This increases the probability of subsequent factors^44^. A flowchart of the simulation system is presented in Fig. 5.

**Fig. 6:**
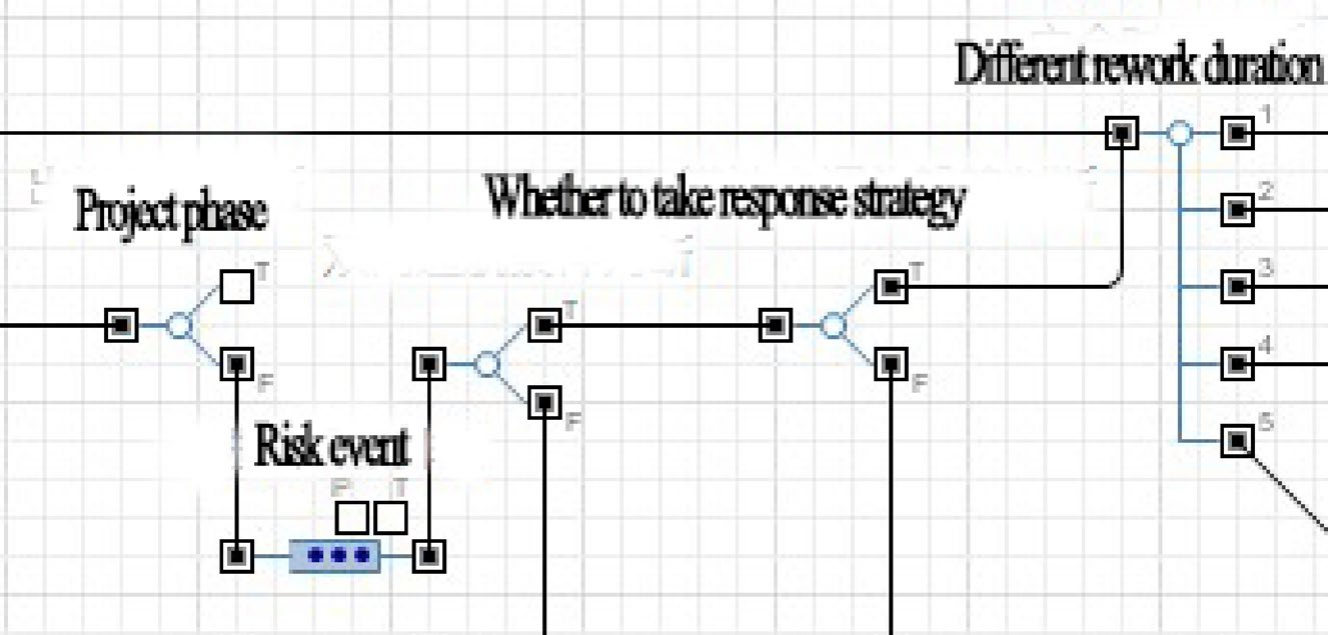
Schematic diagram of risk-factor identification.

In Fig. 6, after the project starts, the simulation model will enter different project phases in sequence, each phase corresponding to a specific set of risks, which will be randomly triggered during the progress of that phase. When a risk occurs, the decision to take risk response strategy can be made based on pre-set instructions, and corresponding to different rework durations. At the same time, any risk event that occurs will have an impact on subsequent events based on the risk relationship matrix, and the probability of subsequent risks occurring will be increased according to the impact weight value. After a risk event ends, whether the project schedule has been reached should be determined firstly. If it has been reached, the simulation of the project ends. If it has not been reached, whether the current phase’s schedule has been reached will be determined. If it has been reached, simulation will proceed to the next phase. If it has not been reached, the simulation of current phase will continue and read the current risk set to simulate the risk event.

**Fig. 7:**
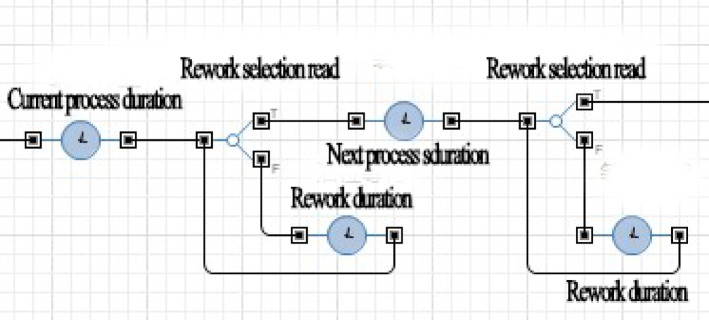
Schematic diagram of process and rework process.

AnyLogic software was used to create the simulation model, this simulation model mainly adopts the process based discrete event modeling paradigm to simulate the sequential workflow and risk events of the project. The process module was used to input each project process. Additionally, risk factors were addressed through probability values assigned by branch flows, and risk response strategies were incorporated into process modules using judgment statements written in Java. When relevant risk factors were identified, statements were made to determine whether response strategies should be implemented. Figures 6 and 7 show partial schematics of the simulation model.

In Fig. 6, when the software simulates a certain project phase, it will automatically read the corresponding risk event and randomly determine whether the event will occur based on the pre input probability of risk occurrence. If an event occurs, a judgment module is used to determine whether to take response strategy, and based on the judgment result, the rework duration is read.

In Fig. 7, when simulating the execution of any process, the software will first read the normal duration and simulate it. Then whether there has been a risk event in this process and the corresponding decision selection will be read, and the rework process according to the rework duration under different decisions will be simulated. After the rework is completed, the software will move on to the next process.

#### Project simulation

##### Input parameter

To obtain the parameters required for the simulation model, this study consulted 45 engineering projects contracted by Chinese contractors in Saudi Arabia over the past 12 years and determined the preliminary data based on the engineering data. Then, an expert panel was meticulously formed, comprising 12 specialists with extensive experience and expertise relevant to international construction projects, particularly in the Saudi Arabian context. The panel’s composition was as follows:

Four project managers from leading Chinese general contracting enterprises, each with over 10 years of experience managing large-scale infrastructure projects in the Middle East.

Three senior managers from local Saudi Arabian construction firms, each with over 10 years of experience managing large-scale infrastructure projects in Saudi Arabia.

Two academic scholars from Saudi universities specializing in construction engineering and project risk management.

Three professors from Chinese universities whose research focuses explicitly on international engineering risk analysis.

The expert group was responsible for reviewing the data and providing modification suggestions to obtain the final parameters. The specific process for obtaining the data was as follows: First, based on the risk factor list in this study, screening was performed using the statistical data of 45 projects in sequence, and the total occurrence frequency of each risk factor was recorded. The frequency of the occurrence of this risk factor in the 45 projects was calculated as the initial incidence. Second, in all projects where this risk occurred, the rework time caused by the risk was recorded in sequence, and the average value was taken as the rework duration corresponding to the risk factor. Finally, based on the risk response strategies presented in this study, the expert group was invited to rate their effectiveness. The scoring system adopted a percentage scale, that is, if an expert believed that the risk response strategy could completely solve the risk, the score was 100. Conversely, if the expert believed that the risk response strategy was ineffective, the score was 0. The expert group scored two aspects: the effect of reducing occurrence incidence and the effect of reducing rework duration, and took the average score as the reduction rate. For example, if the expert group’s average score for the effect of reducing the occurrence rate of a certain risk factor was 30 points, then the initial incidence of that risk factor was multiplied by 70% after adopting the strategies. The specific data are presented in Table 5.

**Table 5:** Simulation model input parameters.

Risk factor	Initial incidence	Rework duration (days)	Incidence after adopting strategies	Rework duration after adopting strategies (days)
*N*_*1*_	0.217	28.4	0.093	8.7
*N*_*2*_	0.135	11.5	0.024	4.4
*N*_*3*_	0.204	13.4	0.107	6.3
*N*_*4*_	0.241	9.6	0.133	7.1
*N*_*5*_	0.172	14.6	0.121	9.7
*N*_*6*_	0.132	12.9	0.094	10.1
*N*_*7*_	0.098	21.6	0.071	12.9
*N*_*8*_	0.182	14.5	0.148	11.1
*N*_*9*_	0.227	8.5	0.167	6.2
*N*_*10*_	0.318	13.4	0.229	11.3
*N*_*11*_	0.254	16.6	0.189	12.5
*N*_*12*_	0.190	8.7	0.141	5.6
*N*_*13*_	0.247	21.4	0.176	16.9
*N*_*14*_	0.115	25.8	0.098	18.2
*N*_*15*_	0.286	18.6	0.155	9.3
*N*_*16*_	0.334	7.6	0.113	3.9
*N*_*17*_	0.094	25.1	0.034	12.5
*N*_*18*_	0.212	14.8	0.135	11.0
*N*_*19*_	0.176	11.9	0.128	9.5
*N*_*20*_	0.246	10.8	0.197	8.7
*N*_*21*_	0.087	8.6	0.064	6.0
*N*_*22*_	0.208	14.8	0.163	11.2
*N*_*23*_	0.156	11.6	0.123	9.7
*N*_*24*_	0.226	18.8	0.189	15.2
*N*_*25*_	0.136	20.7	0.107	16.7
*N*_*26*_	0.217	13.5	0.190	10.1
*N*_*27*_	0.145	9.8	0.121	7.0
*N*_*28*_	0.163	16.4	0.131	12.9
*N*_*29*_	0.248	14.9	0.202	11.8
*N*_*30*_	0.085	7.6	0.063	6.1
*N*_*31*_	0.187	15.5	0.142	11.6
*N*_*32*_	0.126	11.4	0.103	9.4
*N*_*33*_	0.196	14.8	0.161	12.1
*N*_*34*_	0.212	16.6	0.168	13.1

#####  Simulation operation

A mobile workstation was used to execute the simulation model over a single simulation time of 60 months. Java commands were employed to record the output results in each module, and all results were uniformly exported at the end of the model run. To mitigate the impact of random factors on the simulation results, the entire process was preheated thrice before data recording. The model was run 50 times, and the final result was calculated as the average value.

#### Result analysis

##### Comparison of total project progress

**Fig. 8:**
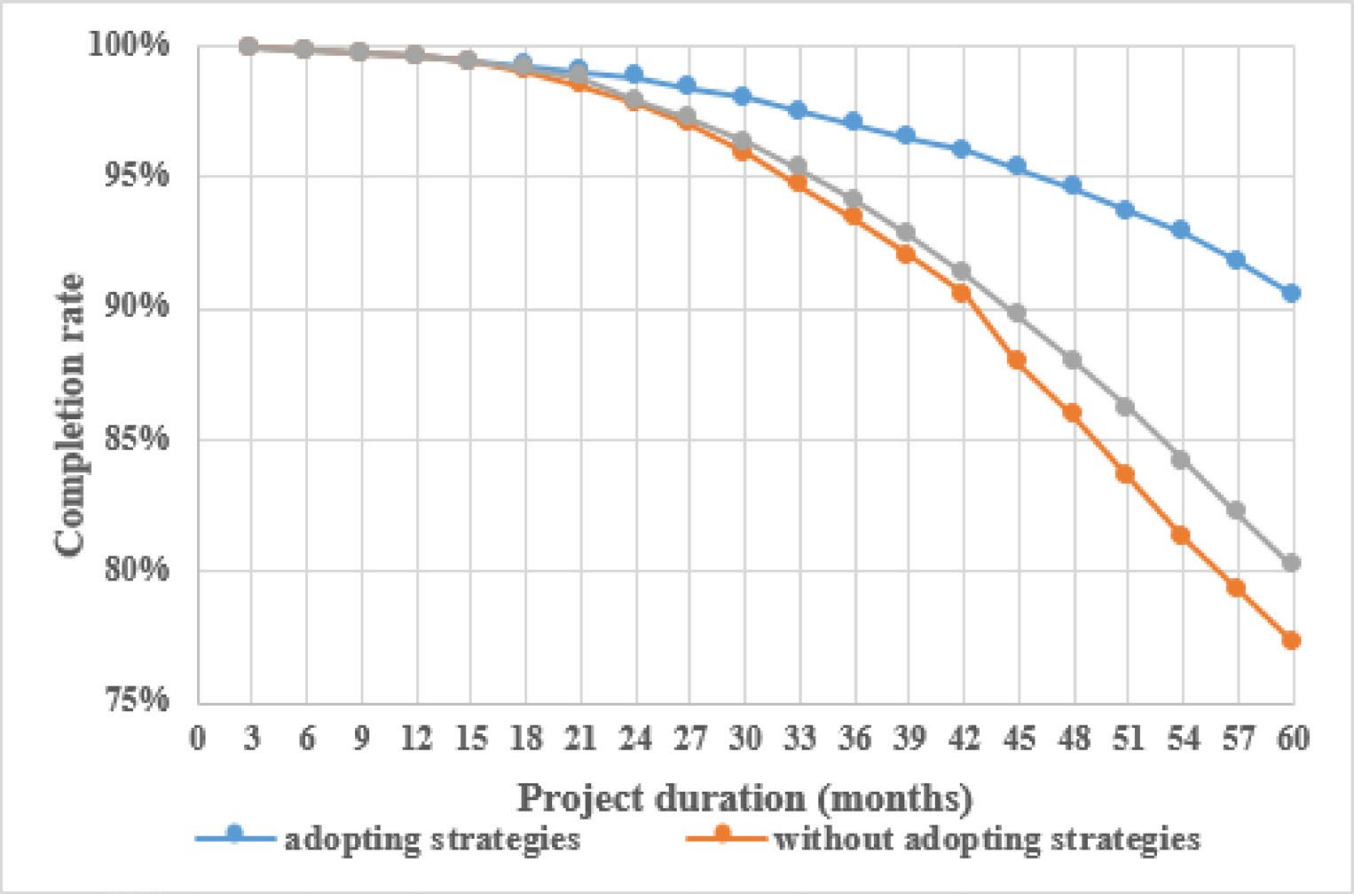
Comparison of total project progress results.

This study conducted simulations under three scenarios: actual conditions, coping strategies, and no coping strategies. The simulation under actual conditions was carried out according to the actual engineering situation, and the risks and rework time were input according to engineering statistical data. Without coping strategies, the risk randomly occurred according to the initial incidence, and the rework duration generated was set according to the initial rework duration. Coping strategies refer to the random occurrence of risks according to their incidence after adopting strategies, and the rework duration is set according to the rework duration after adopting strategies. The purpose of setting these three scenarios was to compare project progress under the three different scenarios to determine the impact of risk factors on the overall construction period and the effectiveness of risk response strategies in controlling the overall construction period. The total progress of the project was obtained for each scenario, as shown in Fig. 8.

Based on Fig. 8, it is evident that without implementing risk response strategies, risk factors significantly impact the project schedule, resulting in a completion rate of approximately 77%. However, after adopting the risk response strategy, the completion rate noticeably increases to 90.5%, indicating that the proposed risk response strategy is relatively effective.

##### Risk factor response efficiency analysis

Two strategies were used to conduct the simulation for each risk factor: pre- and post-emergency treatments. The purpose of the simulation is to compare the actual effects of the two strategies on each risk factor to determine whether the strategies proposed in this paper for each risk factor are reasonable and to determine the most suitable strategy for each risk factor. The corresponding effects of each risk factor were obtained and are listed in Table 5.

The degree of contribution was calculated according to Eq. (3)^45^.

3 $$G\left( i \right)=\frac{{D\left( i \right)+\sum\limits_{{j \in {N_i}}} {D\left( {ij} \right)} }}{{\sum\limits_{{k \in N}} {D\left( k \right)} }}$$

In Eq. (3):

$G\left( i \right)$——Contribution of risk factor *i*;

$D\left( i \right)$——Decrease in rework value of risk factor *i*;

$D\left( {ij} \right)$——Related decrease in risk factor *j* in rework owing to risk factor *i*;

${N_i}$— Set of risk factors associated with risk factor *i*;

The impact factor can be calculated using Eq. (4)^46^.

4 $$F\left( i \right)=\frac{{D\left( i \right)}}{{\sum\limits_{{k \in N}} {D\left( k \right)/N} }}$$

Table 6 demonstrates that, in general, for Type I and Type III risk factors, precontrol had a significantly better effect than post-emergency response, and the average rework decline value was more than 20% higher. This is because the external impact of Type I and Type III risks is higher than the impact they receive, and adopting precontrol strategies can reduce the subsequent impact of these two types of risk. If a post-emergency response is adopted, the risk will spread to other risk areas, and the scope will be larger. Currently, adopting a post-emergency response is not as effective as implementing precontrol strategies. For example, *N*_*5*_ is a Type III risk, and its risk content in existing underground facilities is unclear. The risk factors directly affected by it are *N*_*10*_ and *N*_*18*_, namely, the cost increase due to owner changes and the frequent occurrence of complex geological conditions. If a precontrol strategy is adopted for *N*_*5*_, underground exploration can be conducted in advance to clarify the distribution of underground facilities and create corresponding contingency plans. This can provide sufficient preparation in the event of complex geological conditions and reduce changes caused by collisions or construction difficulties. If a post-emergency response is adopted, when underground problems are discovered during the construction process, the contractor must activate emergency plans, adjust the design and construction plans in response to complex geological conditions, and make changes to the owner. The cost and time required are longer, and the effect is not as good as that of the precontrol strategy. Conversely, the opposite was true for Type II and Type IV risk factors. This indicates that the risk factor classification and countermeasure formulation in this study are accurate and reasonable, leading to effective risk control.

**Table 6:** Risk factor response strategy effect value Statistics.

Number	Risk type	Prior-control			Post-emergency response			Impact factor
		Total rework decreases the value	Associated rework decline value	Contribution	Total rework decline value	Associated rework decline value	Contribution	
*N*_*1*_	II	13.8	0.7	0.083	21.6	1.2	0.099	0.65
*N*_*2*_	III	15.5	8.2	0.175	16.7	9.1	0.182	1.41
*N*_*3*_	III	41.8	22.5	0.281	33.5	17.9	0.248	3.35
*N*_*4*_	III	25.4	13.3	0.204	18.6	10.5	0.172	1.86
*N*_*5*_	III	19.7	10.2	0.186	14.6	8.3	0.155	1.62
*N*_*6*_	I	32.5	15.9	0.229	24.3	11.8	0.193	2.25
*N*_*7*_	III	16.3	8.8	0.182	12.7	7.0	0.165	1.23
*N*_*8*_	IV	22.6	12.2	0.196	23.7	12.8	0.201	1.84
*N*_*9*_	I	25.9	13.7	0.218	21.7	10.6	0.186	1.88
*N*_*10*_	IV	19.3	9.5	0.194	26.9	14.1	0.255	2.09
*N*_*11*_	III	32.8	15.9	0.238	26.3	14.0	0.217	1.96
*N*_*12*_	III	18.6	9.9	0.155	14.3	7.6	0.131	1.42
*N*_*13*_	III	26.8	14.2	0.227	20.8	10.7	0.185	1.85
*N*_*14*_	II	21.6	11.3	0.176	26.9	14.0	0.191	2.13
*N*_*15*_	III	14.6	7.9	0.135	10.3	6.1	0.116	1.12
*N*_*16*_	IV	25.7	19.4	0.235	30.8	22.1	0.251	2.67
*N*_*17*_	IV	63.8	45.1	0.437	81.6	59.5	0.468	5.53
*N*_*18*_	III	33.2	18.4	0.264	29.2	16.5	0.233	3.08
*N*_*19*_	I	35.7	19.6	0.273	28.9	15.8	0.226	2.96
*N*_*20*_	IV	25.7	13.4	0.234	31.6	16.3	0.266	2.83
*N*_*21*_	III	41.7	22.4	0.313	32.8	17.6	0.296	3.45
*N*_*22*_	III	28.8	15.6	0.255	22.9	12.1	0.203	2.17
*N*_*23*_	III	16.9	8.7	0.164	13.4	7.8	0.140	1.47
*N*_*24*_	IV	35.4	20.1	0.292	41.8	23.5	0.325	3.86
*N*_*25*_	III	22.8	12.5	0.191	19.4	10.3	0.171	1.83
*N*_*26*_	III	26.3	14.5	0.214	21.4	11.7	0.203	1.97
*N*_*27*_	IV	15.6	8.6	0.163	21.8	12.0	0.179	2.02
*N*_*28*_	IV	9.8	6.3	0.045	11.2	7.1	0.076	0.57
*N*_*29*_	IV	18.4	9.3	0.188	20.9	11.0	0.213	1.65
*N*_*30*_	III	43.2	28.4	0.311	35.2	24.7	0.289	3.71
*N*_*31*_	I	15.6	12.8	0.179	12.7	9.6	0.093	0.88
*N*_*32*_	IV	30.6	16.2	0.263	36.5	19.1	0.282	2.64
*N*_*33*_	IV	9.7	5.9	0.063	13.7	7.6	0.108	0.63
*N*_*34*_	III	18.5	9.9	0.193	15.2	8.1	0.169	1.68

For some factors, such as *N*_*2*_ and *N*_*31*_, there was no significant difference between the effects of pre- and post-emergency responses. This is mainly because these risk factors are affected to a similar degree by pre- and post-order factors. For these factors, two coping strategies were used as controls. In addition, for risk factors with high contribution and impact, it is important to strengthen risk management and control to avoid risk linkage effects.

### Comparison with previous studies

The identified risk factors in this study, such as extreme weather, cultural differences, and complex approval procedures, align well with the findings of prior research in the Saudi Arabian context^6,8,10^. However, previous studies have primarily treated these risks in isolation, focusing on their individual impacts^3,5^. This study moves a significant step forward by adopting a SNA approach, which reveals the intricate interrelationships and propagation paths among these risks. Our classification of risks into four types (I-IV) based on their network roles provides a more nuanced and systemic understanding than the traditional, list-based risk prioritization commonly found in the literature^13,15^. For instance, while^9^ noted design errors as a critical risk, our network model quantifies its role as a Type I risk, demonstrating its high potential to trigger cascading effects on cost and schedule, that is a dimension previously underexplored.

It is noteworthy that while the general risk landscape in Saudi Arabia has been mapped by researchers from various countries^6,7,11^, the risk perception and impact weights can significantly differ for international contractors. Studies like^10,11^ explored risks from a general international contractor’s viewpoint. Our study specifically addresses the gap for Chinese contractors, whose operational models, supply chain dependencies, and cultural adaptation strategies are distinct. For example, we found that “unstable material supply” (a Type III risk) holds particular severity for Chinese firms who prefer domestic sourcing, a finding that is less emphasized in studies focused on European or local firms^12^. This underscores the critical importance of contextualizing risk management frameworks rather than applying generic models.

The application of SNA in engineering risk management, while growing, often relies on subjective expert judgments for relationship weighting^19,20,22^. A common limitation acknowledged in these studies is the lack of empirical validation of the proposed network models. Our research directly addresses this gap by integrating SNA with a system simulation. The simulation results, which showed a tangible improvement in project completion rate from 77% to 90.5% after implementing our strategies, provide strong and quantitative support for the practical efficacy of the SNA-derived risk control measures. This hybrid methodology strengthens the credibility of the findings beyond what is typically achieved in purely qualitative SNA studies^23,24^.

## Implications, limitations, and future directions

### Implications

On December 8, 2022, the Chinese government and the Saudi government signed the “Implementation Plan for the Joint Construction of “the Belt and Road” and the “Vision 2030” between the two countries” and reached an important consensus on deepening the joint construction of “the Belt and Road” and “Vision 2030.” This plan aimed to jointly promote cooperation between the two countries in infrastructure construction and other aspects. In the future, cooperation between China and Saudi Arabia in municipal engineering projects and other areas will increase. In this context, this study provides a comprehensive list and relationship networks of risk factors that are significant for the risk management of municipal engineering projects contracted by China in Saudi Arabia.

Theoretical Implications. (1) This study demonstrates the efficacy of SNA in moving beyond traditional risk registers towards a more holistic, systemic understanding of risk interdependencies. The identification of four distinct risk types (I, II, III, IV) based on their network positions (e.g., initiators, mediators, terminators) provides a novel typology that transcends the specific risk factors listed. This typology is grounded in network theory, which posits that the role of a node is defined not merely by its attributes but by its relational patterns within the system. We find that preventive strategies are most effective for Type I and Type III risks, while emergency responses suit Type II and Type IV risks. This is not a mere empirical observation but a direct consequence of their network roles. Type I and Type III risks are characterized by high out-degree centrality, and intervening here disrupts entire risk propagation pathways, that is aligning with the principle of managing complex systems by targeting influential nodes. Conversely, Type II and Type IV risks with high in-degree centrality are symptom nodes, and containment and impact mitigation is required to manage them. This provides a theoretically-grounded decision-making framework for resource allocation in risk response, shifting the focus from “what” the risks are to “where” they are located in the network. (2) Saudi Arabia’s political, economic, cultural, and industrial foundations are significantly different from those of China, and there are many uncertainties in project construction. Although previous studies have emphasized the differences in risk factors and project types, they have not paid much attention to municipal engineering projects in Saudi Arabia contracted by China. Research on risk management based on local engineering projects makes it difficult to provide theoretical guidance for China’s contracting of municipal engineering projects in Saudi Arabia. Therefore, the identification of and response to risk factors must be adjusted accordingly. This study provides theoretical guidance for the risk management of multinational engineering projects in this context, as well as a knowledge supplement to risk management theory. (3) The integration of SNA with system simulation represents a methodological advancement. While SNA excels at identifying complex network relationships, its conclusions often rely on subjective expert judgment. The simulation model served as a dynamic scenario analysis tool, testing the causal logic embedded in the SNA-derived network. This mixed-method approach mitigates the subjectivity inherent in SNA and provides a more robust, computational test-bed for evaluating risk response strategies before real-world implementation. This contributes to the methodology of project risk research by demonstrating how qualitative network structures can be operationalized into quantitative, predictive models.

The results of this study have several practical implications. (1) The results of this study will help decision-makers better understand the risk factors of China’s contracting of municipal engineering projects in Saudi Arabia. Based on this, necessary risk response measures can be taken to ensure project success and achieve the 2030 vision goals. (2) The research results can help enterprises cultivate international risk-management teams, respond better to emergencies in complex international engineering projects, enhance the market competitiveness of Chinese engineering contracting enterprises, and increase the bidding and success rates of engineering projects in Saudi Arabia and internationally. Meanwhile, it helps establish a good brand image for Chinese international engineering contracting enterprises and promotes sustainable development. (3) These research results not only provide valuable experience for cooperation between China and Saudi Arabia but also offer lessons for the cooperation and construction of municipal engineering projects between the two countries and other countries. Middle Eastern countries lack skilled resources, rely heavily on foreign nationals, and have a growing demand for new construction projects. The comprehensive list of risk factors and response strategies provides practical references for these countries. (4) We provide a dynamic diagnostic lens for risk management. Instead of a static checklist of country-specific risks, we offer a method to analyze any set of project risks once their interrelationships are mapped. Managers from all kinds of complex international projects can use the provided typology to categorize newly identified risks and apply the corresponding (preventive or emergency) strategy to optimize limited management resources.

### Limitations and future directions

This study has some limitations. First, although this article constructed a comprehensive risk list from past literature and project cases as much as possible, this list may not fully cover the risk factors of municipal engineering projects undertaken by China in Saudi Arabia at different times. The international environment is constantly changing, and the risks faced at different times vary. For example, after COVID-19, cross-border municipal engineering projects have faced more complex issues. In addition, this study selected only one urban road and an underground pipeline project for the case study, which may not fully represent other types of projects. Different types of municipal engineering projects face different risk environments. What’s more, the methodology for classifying risk factors into four types based on degree centrality involves a degree of subjectivity in the threshold determination and ranking process. To address these limitations, future research should focus on building a dynamic risk identification model with empirically validated thresholds and increasing the sample size and types of cases to form a more comprehensive risk analysis framework in time and space.

## Conclusions

This study developed a novel risk analysis framework by integrating Social Network Analysis (SNA) and system simulation to address the complex risk interdependencies in Chinese-contracted municipal engineering projects in Saudi Arabia. The principal findings and contributions are summarized as follows:

Firstly, a comprehensive risk relationship network model was constructed, identifying 34 critical risk factors. By employing SNA, this research moves beyond traditional, isolated risk listings by quantifying the intricate interactions among these factors. A significant methodological contribution is the introduction of a four-type risk classification (I-IV) based on nodes’ network positions and characteristics, utilizing a combination of in and out degree centrality. This typology provides a more nuanced understanding of risk roles within the project ecosystem than conventional prioritization methods.

Secondly, the analysis of a real-world case study led to the development of targeted, type-specific response strategies. The results demonstrate that proactive control measures are most effective for Type I (source) and Type III (influential) risks, while reactive emergency responses are more suitable for Type II (sink) and Type IV (affected) risks. This finding underscores the critical importance of aligning risk mitigation strategies with the intrinsic network properties of each risk, thereby enabling more efficient and effective resource allocation for project managers.

Thirdly, and crucially, the validity of the SNA-derived network and the proposed strategies was rigorously tested through a system simulation model. The simulation results provided quantitative evidence of the strategies’ effectiveness, showing a substantial improvement in the project completion rate from 77% to 90.5%. This simulation step addresses a common limitation in qualitative SNA studies by offering empirical scenario analysis, thereby significantly enhancing the credibility and practical applicability of the findings.

In conclusion, this research contributes to the body of knowledge by providing a validated, network-driven framework for risk management in international construction projects. It offers Chinese contractors a scientifically-grounded tool to navigate the complex risk landscape in Saudi Arabia, with potential applicability to other cross-cultural engineering contexts. For future research, expanding the risk list dynamically and applying this framework to a broader range of project types would further strengthen its generalizability.

## Data Availability

The datasets used and/or analyzed during the current study are available from the corresponding author upon request.
